# A high-throughput, fully automated competition assay to evaluate SARS-CoV-2 neutralizing responses and epitope specificity in clinical samples

**DOI:** 10.1038/s41598-025-94317-2

**Published:** 2025-04-04

**Authors:** Yusuke Atarashi, Jeeeun Kim, Yasuhiro Irino, Masayuki Amano, Kiyoto Tsuchiya, Kenji Maeda, Mari Terada, Noriko Iwamoto, Shinya Shimada, Hiroaki Mitsuya, Masatoshi Yanagida, Yuki Takamatsu

**Affiliations:** 1 Central Research Laboratories, Sysmex Corporation, Kobe, 651-2271 Japan; 2 Department of Clinical Retrovirology, Joint Research Center for Human Retrovirus Infection, Kumamoto University, Kumamoto, 860-8556 Japan; 3 AIDS Clinical Center, Center hospital of the National Center for Global Health and Medicine, Tokyo, 162-8655 Japan; 4 Refractory Viral Diseases, National Center for Global Health and Medicine Research Institute, Tokyo, 162-8655 Japan; 5 Department of Disease Control Center, Center Hospital of the National Center for Global Health and Medicine, Tokyo, 162-8655 Japan; 6 Japan Community Healthcare Organization, Kumamoto General Hospital, 866- 8660 Kumamoto, Japan; 7 Experimental Retrovirology Section, HIV and AIDS Malignancy Branch, National Cancer Institute, National Institutes of Health, 20892-1868 Bethesda, MD USA; 8 Division of Clinical Sciences, Kumamoto University Hospital, 860-8556 Kumamoto, Japan; 9 Division of Antiviral Therapy, Joint Research Center for Human Retrovirus Infection, Kagoshima University, Kagoshima, Japan 890-8544

**Keywords:** SARS-CoV-2, High throughput testing, Antibody test, Neutralizing activity, SARS-CoV-2, Viral infection, Viral infection, Infection, Antimicrobial responses, Immunochemistry, Assay systems

## Abstract

Coronavirus disease-2019 (COVID-19) remains a critical global health concern. We developed a fully automated, high-throughput competition immunoassay to elucidate how epitope recognition on the severe acute respiratory syndrome coronavirus 2 (SARS-CoV-2) spike receptor-binding domain (RBD) correlates with neutralizing activity. Analysis of clinical samples from both SARS-CoV-2-infected and vaccinated individuals revealed that vaccination elicits significantly higher antibody titers across multiple S1 subunit epitopes compared to natural infection. Notably, median antibody levels against the receptor-binding motif (RBM) exceeded 50% in both cohorts, highlighting the RBM as a key target for antibody induction irrespective of immune origin. Furthermore, the strongest correlation with neutralizing activity was observed for antibodies directed against the broader S1 subunit, indicating that epitopes outside the RBM also contribute to neutralization. These findings underscore the importance of both RBM- and non-RBM-directed antibodies in effective immune defense against SARS-CoV-2. Our assay enables large-scale, reliable quantification of neutralizing antibodies and provides critical insights for developing improved diagnostic antigens and vaccine strategies aimed at eliciting robust, multi-epitope immune responses.

**Supplementary Information:**

The online version contains supplementary material available at 10.1038/s41598-025-94317-2.

## Introduction

As of July 2024, the coronavirus disease-2019 (COVID-19) outbreak, declared a global pandemic by the World Health Organization, has reportedly accounted for over 7.54 million deaths worldwide (https://data.who.int/dashboards/covid19/cases)^1,2^. It is caused by the severe acute respiratory syndrome coronavirus-2 (SARS-CoV-2), an RNA virus that has undergone rapid evolution, changing its genomic sequence to acquire distinct characteristics such as increased transmissibility, enhanced severity, and ability to evade the immune system^3^. SARS-CoV-2 mutations resulting in variants with elevated transmission rates compared to earlier variants have been classified as variants of concern (VOCs). Five independent VOCs, namely Alpha, Beta, Gamma, Delta, and Omicron, have been identified to date^4^.

Vaccines play a crucial role in the combat against COVID-19^5–7^. However, vaccines need to be continually modified for sustained protection due to rapid virus evolution, yielding new strains^8,9^. The fundamental principle of most currently available COVID-19 vaccines is to inhibit the interaction between the virus’s spike protein receptor-binding domain (RBD) and human angiotensin-converting enzyme 2 (ACE2). Therefore, immunogens capable of potently stimulating increased production of neutralizing antibodies (nAbs) against epitopes are urgently needed. Efforts to understand nAb responses to SARS-CoV-2 have significantly accelerated vaccine development^10^. nAbs are closely linked to the antigen-binding region^11^. Therefore, elucidating the relationship between the binding region of nAbs and their neutralizing activity is essential to assess their neutralizing potential accurately. However, large-scale clinical sample analysis is complex due to the lack of high-throughput analysis tools^12,13^.

Various quantitative- or qualitative-serologic assessments have been developed to evaluate antibody responses, each characterized by its sensitivity and performance. Enzyme-linked immunosorbent assays (ELISAs), for instance, are widely used for their high sensitivity and specificity but are limited by their lower throughput and longer processing times. Lateral flow assays (LFAs) offer rapid results and ease of use but often suffer lower sensitivity than ELISAs^14^. Of note, these serum antibody tests do not necessarily predict the presence of neutralizing antibodies or protection from disease^15^. Neutralization assays, considered the gold standard, directly assess the ability of antibodies to inhibit viral infection but are labor-intensive and unsuitable for high-throughput screening^16^. These limitations underscore the need for a high-throughput, automated system to assess neutralizing antibody responses accurately.

**Fig. 1:**
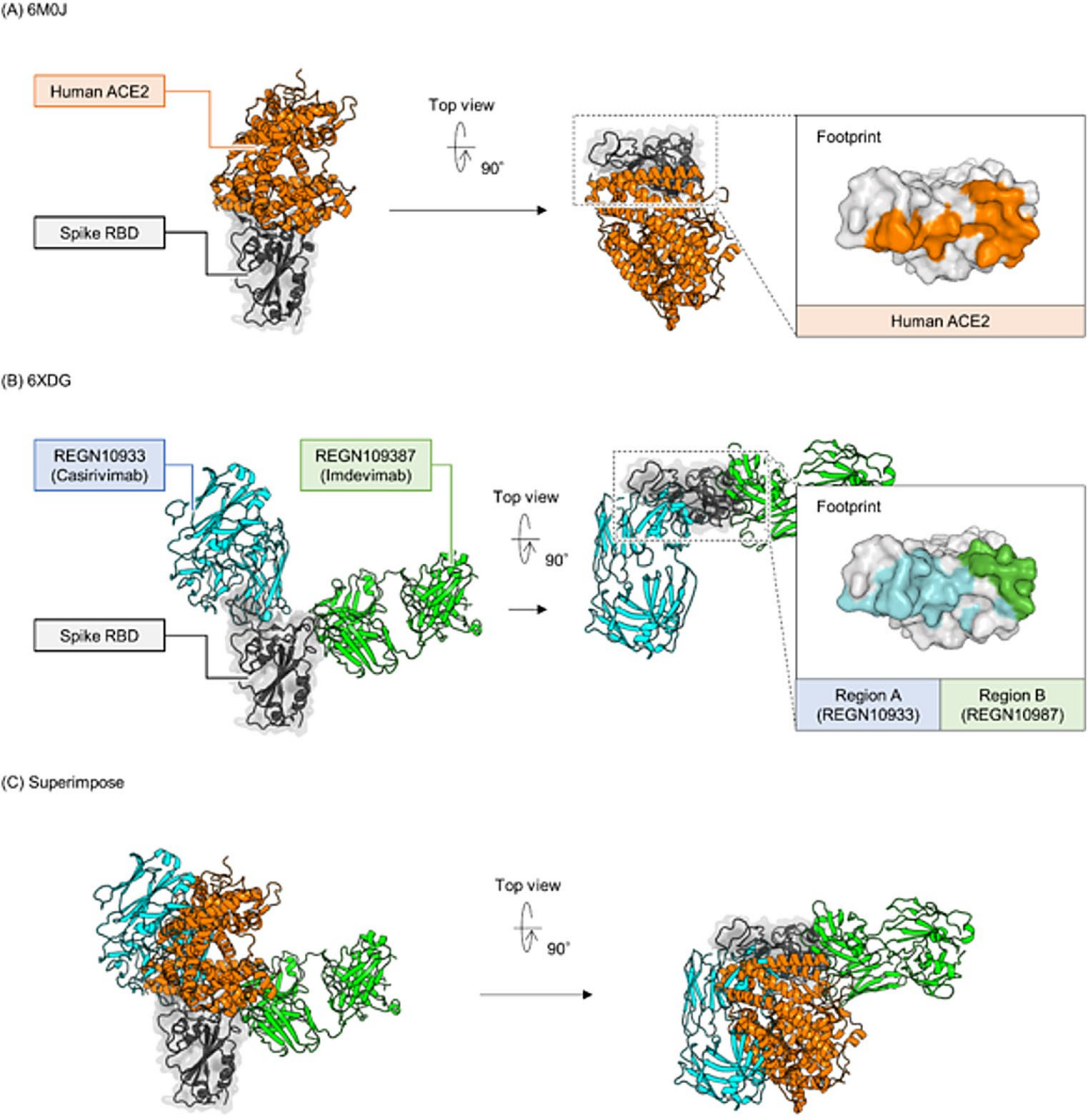
Crystal structure of the SARS-CoV-2 RBD in complex with ACE2 or neutralizing antibodies.The RBD surface is colored to highlight interacting residues identified using Discovery Studio 2022. Two distinct regions, designated as Region A and Region B, represent the non-overlapping epitopes targeted by REGN10933 (Casirivimab) and REGN10987 (Imdevimab), respectively, which cover different sides of the RBM. (**A**) RBD bound to human ACE2 (PDB 6M0J), with ACE2-interacting residues in orange. (**B**) RBD bound to two neutralizing antibodies (PDB 6XDG), with interacting residues in green and cyan. (**C**) Superposition of the complexes, illustrating how these regions are spatially separated.

The HISCL system is a fully automated chemiluminescent sandwich-based platform that affords high sensitivity, specificity, and reproducibility^17^. In this study, we extended its capabilities by incorporating a competition assay with two clinically approved monoclonal antibodies, REGN10933 (Casirivimab) and REGN10987 (Imdevimab). These antibodies target complementary, non-overlapping epitopes within the receptor-binding motif (RBM), effectively covering different surfaces of the ACE2-binding interface while minimizing mutual interference (Fig. 1). By applying this system to clinical samples from virus-infected and vaccinated individuals, we aimed to elucidate how specific antibody-binding profiles correlate with neutralizing activity. Assessing naturally acquired versus vaccine-elicited immune responses under real-world conditions provides critical insights into how epitope-specific antibodies influence overall neutralization, thereby informing more effective diagnostic and therapeutic strategies.

## Materials and methods

### Clinical samples and ethics

This study included COVID-19 convalescent individuals^18^ and COVID-19 vaccinated individuals without a previous history of COVID-19 in earlier studies conducted at the National Center for Global Health and Medicine (NCGM), Tokyo, Japan. (approval nos. NCGM-G-003536 and NCGM-G-004176). This study was conducted under the Declaration of Helsinki and approved by the Medical Research Ethics Committee of NCGM and Sysmex Corporation (approval nos. NCGM-S-004403 [NCGM] and 2021 − 107 [Sysmex]). Informed consent was obtained from all patients. As per the Ethical Guidelines for Medical and Health Research Involving Human Subjects, information on this study has been publicly disclosed, and the participants were allowed to withdraw their consent at any time. Therefore, the ethics committee waived the need for written informed consent from enrolled patients.

In total, 300 clinical samples, consisting of sera from 150 COVID-19 convalescent individuals without any vaccination^19,20^ and 150 individuals who received 3rd booster-dose of ancestral monovalent BNT162b2 without a primary history of COVID-19^21^, were collected. Specimens from infected individuals were collected between June 12, 2020, and November 25, 2020. The median (IQR) duration from symptom onset to sample collection was 59 (36–101), except for 18 individuals whose onset date was missing. The convalescent group did not include reinfection cases. Specimens from vaccinated individuals were collected between December 15, 2021, and December 20, 2021. The median (IQR) duration from booster dose (3rd BNT162b2 vaccination) to sample collection was 13 (12–14). The in vitro neutralizing activity cell-based assay using live clinically-isolated SARS-CoV-2 ancestral variant was conducted as previously described as reference test^17,19,20^.

### Production of recombinant antibodies for competition assay

Two antibody structures used in the competition assay were obtained from the Protein Data Bank (PDB). Both have resolved co-crystal structures with the receptor-binding domain (RBD) (PDB ID: 6XDG)^22^. Based on these structural data, we used the Analyze Protein Surface function in Discovery Studio 2022 to identify residues that interact with the antibodies (Fig. 1). The *VH* and *VL* gene sequences were also retrieved from the PDB. We then designed the competing antibodies by grafting each *VH* and *VL* sequence onto the constant region of mouse IgG2a (kappa) (accession numbers: P01863 and P01837). Human antibodies were designed as a control by grafting onto a constant region of human IgG1 (kappa) (accession: P0DOX5 and P01834). Each sequence was cloned into a pcDNA3.4 vector (Thermo Fisher Scientific, Waltham, MA, USA) and transfected into Expi293 cells (Thermo Fisher Scientific) according to the manufacturer’s protocol. Six days post-transfection, the supernatants were collected, and recombinant antibodies were purified using the HiTrap Mabselect SuRe column (Cytiva, Marlborough, MA, USA) and Superdex 200 increase 10/300 GL column (Cytiva).

### Assay description

SARS-CoV-2 antibody competitive immunoassays were performed using the HISCL system. The HISCL system is a fully automated system based on the chemiluminescent sandwich method. For this assay, serum or plasma samples were mixed with a competitive antibody. This mixture was subsequently incubated with SARS-CoV-2-specific recombinant antigens bound to magnetic beads. After separating the bound and free components, the antigen-antibody complex was incubated with an alkaline phosphatase-conjugated antibody against human IgG to form a sandwich immunocomplex. Following the second separation, a luminescent substrate was added to the solution for luminescence measurements. The entire reaction was carried out at a constant temperature of 42 °C, and the luminescence intensity was measured within 17 min. An overview of this competition-based assay is illustrated in Fig. S2. If sample-derived antibodies bind near the epitope recognized by the competing antibody, the competing antibody blocks their binding, thereby reducing the signal. The antibody titer corresponding to each region was calculated using the following formula:

(titer without competitive antibodies) – (titer with competitive antibodies).

### Statistical analyses

All statistical analyses were conducted using R version 4.3.2. Results are expressed as the median (range) and 95% confidence interval (CIs). The ratio of antibody titers in each region to the S1 antibody titer was calculated, limiting to samples with an S1 antibody titer ≥ 20 BAU/mL, and statistical analysis was conducted. Correlation analysis was performed using Spearman’s rank correlation test. Statistical significance was set at *p* < 0.05.

## Results and discussion

We developed a fully automated HISCL system for high-throughput detection of binding site–specific antibodies in patient specimens. By measuring region-specific antibody titers, this system enables detailed analysis of how individual epitopes contribute to the clinical immune response. We defined two distinct regions, designated Region A and Region B, within the RBD of the SARS-CoV-2 spike protein. These regions were determined based on the binding footprints of REGN10933 and REGN10987, respectively, which predominantly target different sides of the RBM (Fig. 1). This design allows separate quantification of antibodies directed against each region, providing deeper insight into epitope-specific immune responses. We verified the competition effect by conducting a competition assay with a human monoclonal IgG antibody sharing the same variable region as the competing antibody, which produced > 80% signal reduction (Fig. S1).

**Fig. 2:**
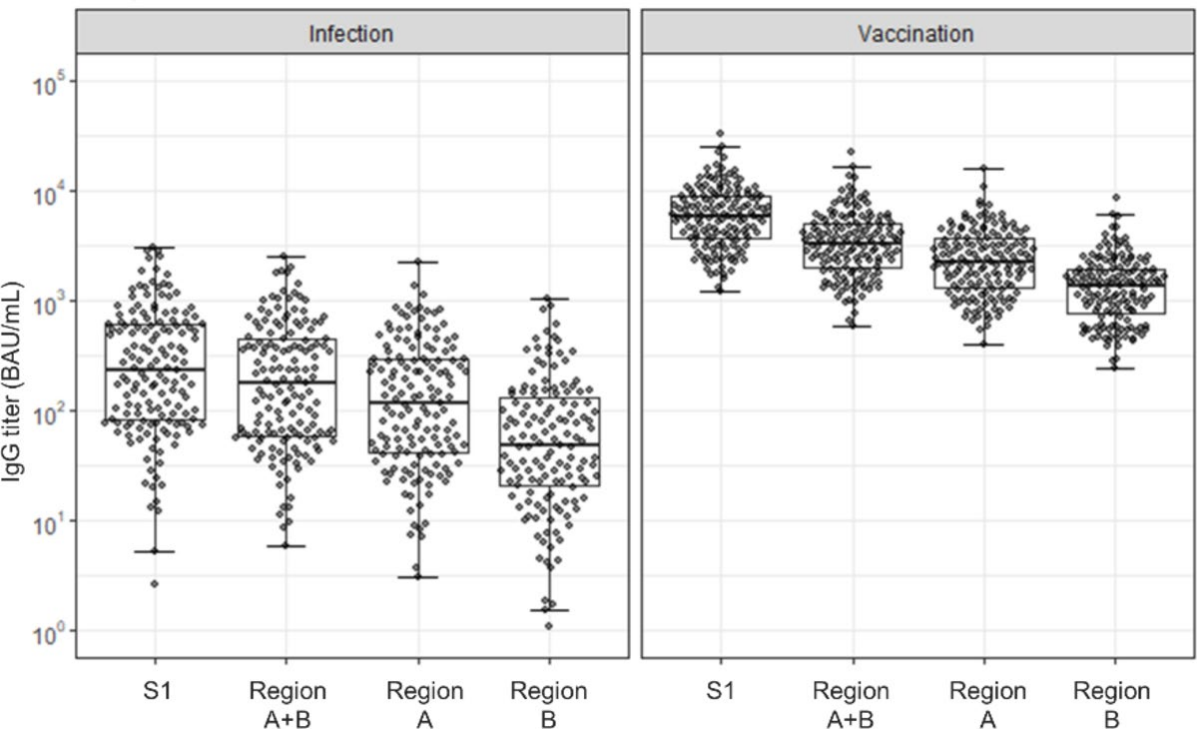
Epitope-specific antibody titers. Antibody titers were calculated as the difference between measurements obtained without and with competitive antibodies. The x-axis displays four regions: S1 (the Spike S1 subunit), Region A + B, Region A, and Region B (Regions A and B are detailed in Fig. 1). (**A**) Antibody titers in samples from virus-infected individuals. (**B**) Antibody titers in samples from vaccinated individuals.

Initially we evaluated region-specific antibody titers in sera from vaccinated and virus-infected individuals (Fig. 2). Overall, the vaccinated group displayed higher antibody titers across all assessed regions than the convalescent group. This result suggests that three vaccine doses cause more efficient antibody induction than antigen exposure due to a natural viral infection.

**Fig. 3:**
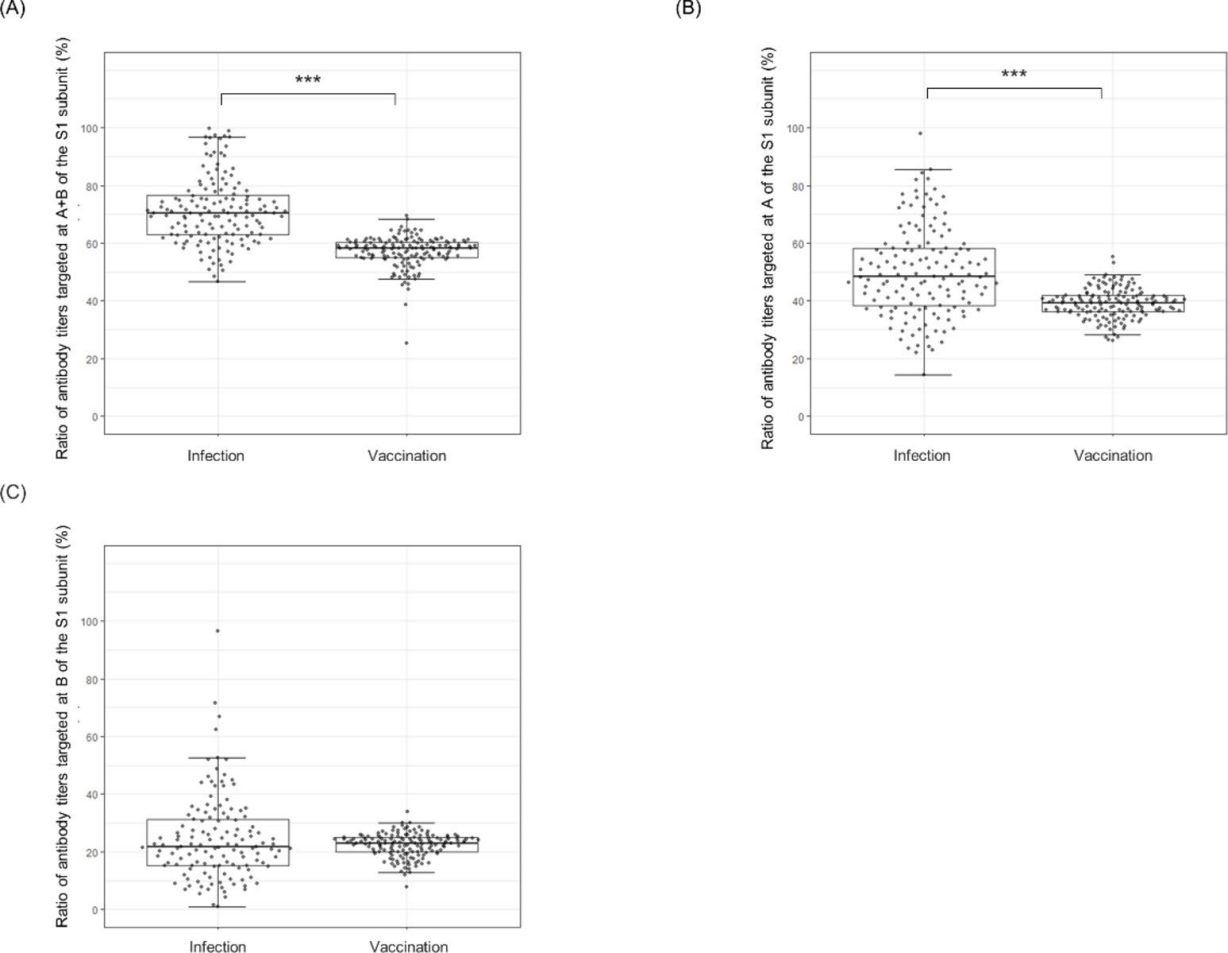
Ratio of antibody titers targeting defined regions of the S1 subunit. The y-axis in each panel represents the percentage of total S1-targeting antibodies that bind to the indicated region(s). P-values were calculated using the Wilcoxon–Mann–Whitney test with R (***p < 0.001). (**A**) The ratio of antibody titers targeted at A + B of the S1 subunit. (**B**) The ratio of antibody titers targeted at A of the S1 subunit. (**C**) The ratio of antibody titers targeted at B of the S1 subunit.

Next, we focused on samples with an S1 antibody titer ≥ 20 BAU/mL to assess the distribution of antibodies targeting the RBD (Fig. 3). The median for both virus-infected and vaccinated individuals exceeded 50% in regions A and B (Fig. 3A), indicating that the RBM is a major target for SARS-CoV-2 antibody induction, regardless of the infection or vaccination status.

To evaluate the antibody titer ratio of A + B in S1 and A in S1, we revealed a trend in which infected individuals had significantly higher ratios than vaccinated individuals (Fig. 3B). This suggests that vaccination with the ancestral monovalent BNT162b2, which encodes a stabilized full-length spike protein^23^, might promote a broader epitope recognition compared to natural infection, resulting in more uniform coverage of both RBM and non-RBM epitopes.

**Fig. 4:**
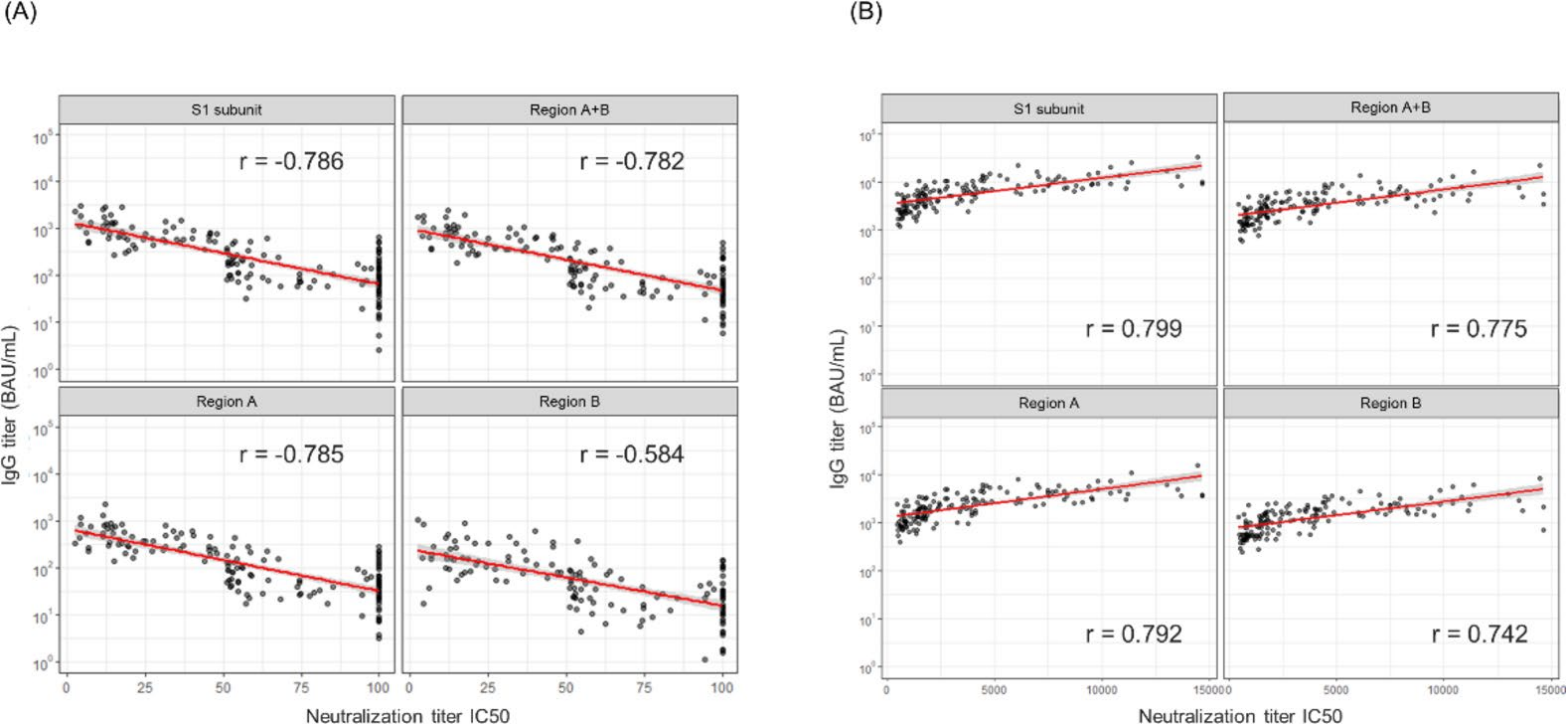
Correlation between the antibody titer and neutralizing activity. The red and gray areas indicate the regression lines and 95% confidence intervals, respectively. Spearman’s rank correlation coefficient was calculated using R. (**A**) Correlations in the infected samples. (**B**) Correlations in the vaccinated samples.

We further investigated the relationship between the activity of nAbs and epitope regions targeted by the antibodies. Spearman’s rank correlation coefficient revealed the strongest correlation between the antibody levels of the S1 subunit and neutralization activity in both the virus-infected and vaccinated individuals (Fig. 4A and B). Interestingly, focusing solely on a narrow antibody-binding domain did not necessarily enhance the correlation with neutralization. While RBM-targeting antibodies directly block ACE2 binding and exhibit potent neutralizing activity^11^, our findings suggest that non-RBM antibodies may also contribute to neutralization through indirect mechanisms.

Although several previous studies have reported such effects^24–26^, our study provides additional evidence from clinical samples, highlighting the importance of targeting multiple epitopes for effective SARS-CoV-2 neutralization. Future studies should investigate (i) cross-neutralization, (ii) targeting the N-terminal domain (NTD), (iii) targeting the S2 subunit, (iv) preventing S1 shedding, (v) steric hindrance, and (vi) preventing conformational changes to further elucidate the roles of non-RBM antibodies. Establishing an antibody screening system using HISCL could facilitate the discovery of such antibodies, contributing to the development of next-generation therapeutics.

Currently, the ACE2 competition assay is the primary platform for determining the neutralizing activity of antibodies in clinical use as competitive virus neutralization tests (cVNTs) against SARS-CoV-2^27^. However, this method is limited to detecting only ACE2-blocking antibodies, overlooking those targeting other key epitopes. In contrast, our system employs two monoclonal antibodies that target complementary, non-overlapping epitopes within the RBM, enabling a more refined characterization of antibody responses. This approach can reveal how specific viral mutations might diminish antibody binding to one epitope while sparing the other, providing valuable insights into immune escape mechanisms. Additionally, using clinically approved antibodies (Casirivimab and Imdevimab) links our results directly to therapeutic strategies, illustrating how serum-derived antibodies may compete with or complement these treatments in real-world scenarios.

In conclusion, we developed a novel high-throughput system using a fully automated immunoassay to detect the antibody epitopes in specimens, focusing on two regions of the SARS-CoV-2 RBD. Clinical sample analysis revealed that the vaccinated individuals exhibited higher antibody titers across all epitope regions than the virus-infected individuals. Our findings suggest that antibodies binding to regions other than RBM also indirectly contribute to the neutralizing activity. Overall, our highly automated immunological assay system can facilitate the design of novel diagnostic antigens tailored to the specific objectives of antibody testing in clinical settings.

## Electronic supplementary material

Below is the link to the electronic supplementary material.

Supplementary Material 1

Supplementary Material 2

## Data Availability

No publicly available repositories or databases are suitable for the current data submission. All data supporting the results of this study are available in the article. They can also be obtained from the corresponding author, YT, upon reasonable request.

## Abbreviations

ACE2: Angiotensin-converting enzyme 2
COVID-19: Coronavirus disease-2019
nAbs: Neutralizing antibodies
RBD: Receptor-binding domain
RBM: Receptor-binding motif
SARS-CoV-2: Severe acute respiratory syndrome-coronavirus-2
VOCs: Variants of concern
