# Supplementary figures and images for: A high-throughput, fully automated competition assay to evaluate SARS-CoV-2 neutralizing responses and epitope specificity in clinical samples
Source: Sci Rep. 2025 Apr 4;15:11589. doi: 10.1038/s41598-025-94317-2 (PMC11971398; doi:10.1038/s41598-025-94317-2)

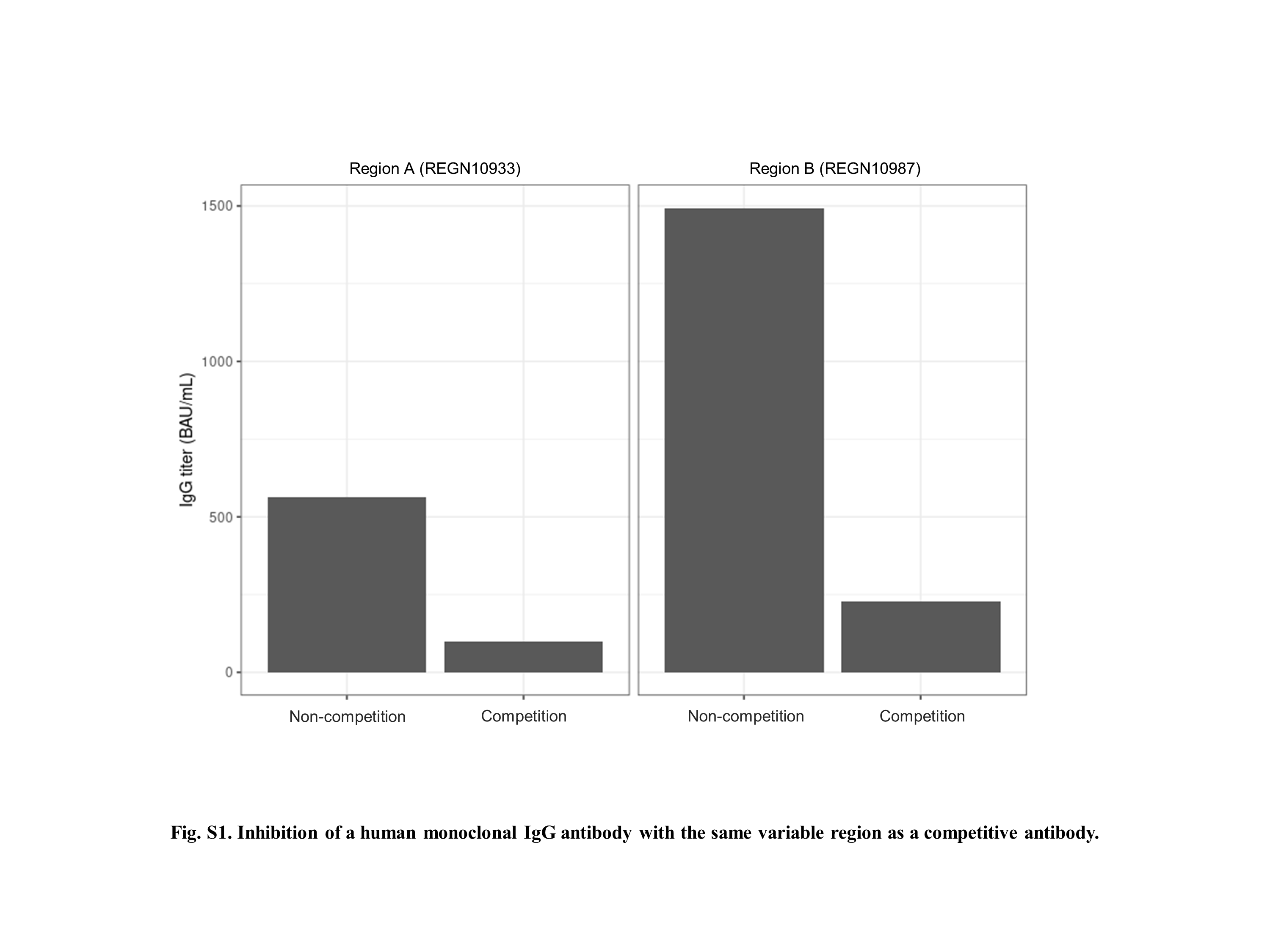

Supplement: Supplementary file 1 — Supplementary Material 1 [file 41598_2025_94317_MOESM1_ESM.tif]

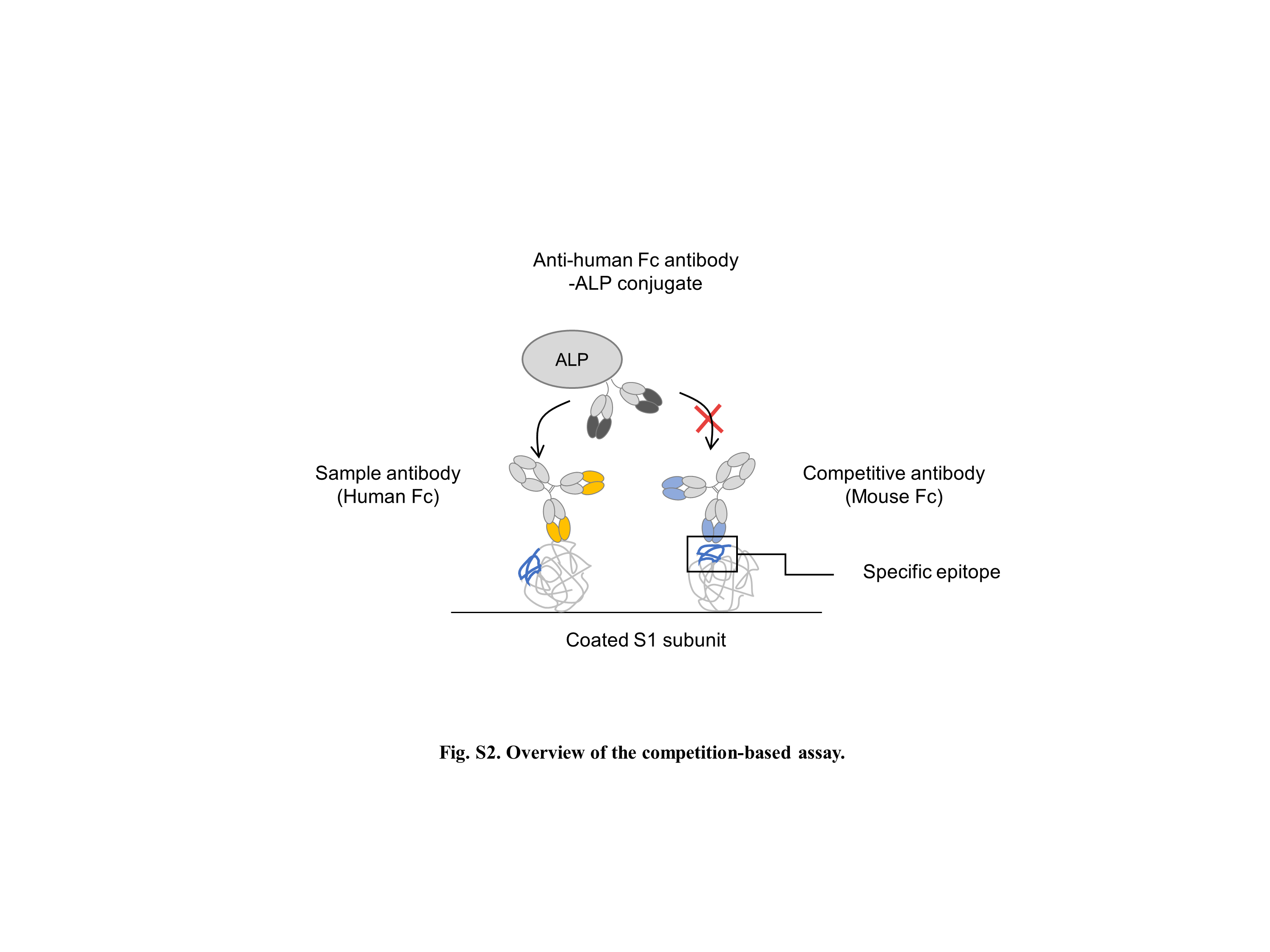

Supplement: Supplementary file 2 — Supplementary Material 2 [file 41598_2025_94317_MOESM2_ESM.tif]
